# Unmanned Vehicles’ Placement Optimisation for Internet of Things and Internet of Unmanned Vehicles

**DOI:** 10.3390/s21216984

**Published:** 2021-10-21

**Authors:** Ana-Maria Dragulinescu, Simona Halunga, Ciprian Zamfirescu

**Affiliations:** Telecommunications Department, Faculty of Electronics, Telecommunications and Information Technology, University Politehnica of Bucharest, 061071 Bucharest, Romania; simona.halunga@upb.ro (S.H.); ciprian_zamfirescu@yahoo.com (C.Z.)

**Keywords:** algorithm, Internet of Unmanned Vehicles, link budget, LoRa/LoRaWAN, optimisation, path loss, Wireless Sensors Networks, UAV, USV, Wi-Fi

## Abstract

Currently, the use of unmanned vehicles, such as drones, boats and ships, in monitoring tasks where human presence is difficult or even impossible raises several issues. Continuous efforts to improve the autonomy of such vehicles have not solved all aspects of this issue. In an Internet of Unmanned Vehicles (IoUV) environment, the idea of replacing the static wireless infrastructure and reusing the mobile monitoring nodes in different conditions would converge to a dynamic solution to assure data collection in areas where there is no infrastructure that ensures Internet access. The current paper fills a significant gap, proposing an algorithm that optimises the positions of unmanned vehicles such that an ad hoc network is deployed to serve specific wireless sensor networks that have no other Internet connectivity (hilly/mountainous areas, Danube Delta) and must be connected to an Internet of Things (IoT) ecosystem. The algorithm determines the optimum positions of UV nodes that decrease the path losses below the link budget threshold with minimum UV node displacement compared to their initial coordinates. The algorithm was tested in a rural scenario and 3rd Generation Partnership Project (3GPP), free space and two-ray propagation models. The paper proposes another type of network, a Flying and Surface Ad Hoc Network (FSANET), a concept which implies collaboration and coexistence between unmanned aerial vehicles (UAVs) and unmanned surface vehicles (USVs) and several use cases that motivate the need for such a network.

## 1. Introduction

Unmanned aerial vehicles or drones proved to be key enablers and productivity promoters [1] for a wide range of domains, such as environmental monitoring, precision agriculture, smart cities and disaster management, complementing all the IoT technologies currently in use. Their practical utility comes from the ability to perform real-time tasks in hard-to-reach areas [2]. UAVs have different sizes and, thus, capabilities, as the dimensions of a drone are related to its equipment [1].

With the advent of the Internet of Things and with the use of drones not only restricted to industry, new concepts called “Internet of Drones“ (IoD) or “Internet of Unmanned Aerial Vehicles“ (IoUAV) arose, designating a layer-based network architecture used to connect a high number of drones, also coordinating their access to the controlled airspace [3,4]. One of the challenges of such an architecture design relates to airspace awareness [5]. In addition, another issue consists of navigation while forwarding data between different nodes [3,6] when flying vectors are usually in a non-line-of-sight (NLoS) scenario [5]. The drones have been proved to leverage the topologies and performances of the networks themselves [6,7].

In IoD real-life deployment, the drones will be heterogeneous from the point of view of transmission technologies and protocols, as they can perform different tasks. UAVs networks, usually called Flying Ad Hoc NETworks (FANET), not only provide usual network services but also for task/objective fulfilment or mission accomplishment, as they are capable of performing these tasks [1,8]. Such networks bring several benefits: mobility, on-site image processing, tri-dimensional (3D) mapping, etc. In addition, on-demand deployment is possible, thus making new and expensive infrastructure unnecessary if the network is only to be used for a short time [9]. Currently, FANET represents a component of the Vehicular Ad Hoc Network (VANET) which, in turn, is included in the Mobile Ad Hoc Network (MANET). Unlike MANET and VANET, in FANET, the node density is significantly lower [10].

UAV networks’ capabilities can be improved by using USVs (Unmanned Surface Vehicles) and surface ad hoc networks (SANET) [11]. Moreover, one important challenge related to UAVs’ reliability refers to their limited battery life. Consequently, when a Ground Control Station (GCS) [7] or a Ground Recharging Station (GRS) are not available due to distance or other factors (weather, for instance), USVs are good candidates to assure efficient, on-time battery charging, acting as mobile recharging stations or as landing points [12]. Additionally, UAV battery recharge scheduling algorithms were proposed [13]. UAVs can also integrate these algorithms such that the charging schedule is optimised in terms of energy and distance to the USV charging station.

UAVs, in exchange, can assist USVs’ navigation by utilising the possibility to gather environmental data from a 3D space [14]. In addition, UAVs can extend the range of USVs’ communication.

In addition to energy extension cooperation, in large-scale IoT architectures, UAVs and USVs can act both as data and image collectors and as network relays [15] and aggregators, as unmanned vehicles have the great advantage of mobility in comparison to the usual network equipment. Numerous researchers provided algorithms that control UAVs’ formation in order to increase the networks’ performances [7,16,17,18,19], but currently, no algorithm that envisions the formation of a UAV through UAV–USV cooperation is available. For data collection, Wireless Sensors Networks (WSNs) can be deployed in this UAV–USV cooperation to monitor and collect environmental data. WSNs are widely used for environmental monitoring in many fields, such as agriculture, disaster management (e.g., floods) and water monitoring, and a real problem resides in their further connection to the Internet infrastructure when they are deployed in isolated areas. Thus, UAVs can perform as data collection nodes to improve the energy consumption performance of WSNs and their coverage. In addition, the improvements consist of increased data transmission efficiency and lifetime [20]. On the other hand, when monitoring water quality, for example, WSNs may be deployed on buoys floating on the water surface and UAVs can collect their data as in [21] through the use of communication technology as such LoRa/LoRaWAN. The use of buoys, though, may support the increase in network coverage for WSNs found onshore, but usually, the interval between consecutive data transmissions is reduced, and the deployment of many such buoys would be expensive, as special protection enclosures are required. A more efficient approach would be to employ USVs and UAVs which are mobile and can serve more areas on demand. In addition, the adoption of a single communication technology determines the low scalability of the system to other applications.

In the literature, when developing algorithms for UAV formation or USV formation, the propagation aspects (environment, different types of channels and propagation models) are usually neglected. If the employment of UAVs as access points was previously studied [22], the usage of USV as relays is less clear. When referring to SANET, the works found in the literature approach the large-scale maritime communications on different specific standards, such as ITU-R M.1842-1 [23] dedicated for very high-frequency (VHF) communications between industrial ships, but small-scale applications, such as those on rivers and lakes, are less represented. The usage of USVs as access points is motivated by the specific characteristics of some areas close to water environments and by many applications in regions transpassed by important rivers, such as the Danube (in Europe). Other regions of interest are those close to lakes that have a significant surface area. VHF communications would require very large antennas which are not affordable for small vehicles such as UAVs and USVs. Consequently, this paper intends to provide an algorithm for unmanned vehicles’ (UVs, including UAVs and USVs) formation, such that communication is assured along a GW–UVs–WSN (Gateway–Unmanned Vehicles–Wireless Sensors Network) communication chain, where Wi-Fi communication is employed for quick data transfer, whereas LoRa/LoRAWAN is employed for non-critical, low-throughput data transfer. Both technologies operate in unlicensed spectra and the necessary equipment to deploy them is miniaturised. The algorithm must determine the optimum position of each UV node such that they become Wi-Fi access points. Then, they are capable of fast-forwarding data from a wireless sensor network (WSN) to a gateway (GW). Based on the optimum positions computed through the algorithm, the equipment (sensors, cameras) and the performances of unmanned vehicles (in particular, UAVs and USVs), afterwards, a Cloud Center can assign different monitoring tasks to the UVs, their role being two-fold: data forwarders and monitoring data collectors.

Concerning the contribution of the paper:

- We highlight the importance of UAV and USV networks, and we propose the concept of Internet of Unmanned Vehicles over the concept of Internet of Drones or Internet of Unmanned Aerial Vehicles, which were narrower. We emphasise the current state of the art and how our proposal goes beyond this.
- We propose an architecture comprising unmanned vehicles and a network of UAVs and USVs (UVs) that both assure reliable communication and fulfil tasks assigned by a Cloud Center (CC).
- We offer the background for the communication link optimisation from the point of view of propagation.
- We propose a new method for reducing the searching space for optimum position problems, namely the great circle path, and we implement an algorithm that determines the closest UV nodes to specific points on this path.
- We propose and implement an algorithm for determining the optimal positions of the UVs such that the communication between the gateway and a target WSN is assured by higher throughput, lower range communication technology. Based on those positions, the Cloud Center can adaptively assign tasks to the UVs.
- We mention the potential use cases of the proposed architecture and algorithm when LoRa/LoRaWAN communication technology is employed and, respectively, when LoRa/LoRaWAN together with Wi-Fi cellular communications is used.
- We highlight the future directions and the open challenges.

The remainder of the paper is as follows: Section 2 presents the state of the art on UAV and USV networks and on the advancements in improving the communication between them in comparison with the approach proposed in this paper. In Section 3, the components of the proposed UAV–USV–WSN network are described, and the proposed Internet of Unmanned Vehicles architecture is depicted. Section 4 briefly presents the context in which the approach is necessary. In Section 5, we introduce the theoretical background and the propagation concept and relevant parameters. In addition, we present the air-related channels and water-related channels that model the path losses in different scenarios. Section 6 describes the great circle path method and the algorithm that derives from it and presents the integration of the practical communication optimisation measures in the proposed position optimisation algorithm. In Section 7, the testing and the performance of the algorithm are emphasised. Section 8 presents the particular use cases of the proposed architecture and algorithm when LoRa/LoRaWAN/Wi-Fi communication technologies are used. Finally, Section 9 concludes the paper, highlights the future perspectives and underlines the open challenges.

## 2. Unmanned Aerial and Surface Vehicle Networks: Applications and Advancement in Their Optimisation

Combined UV networks cover a great area of application, from smart agriculture, smart cities and smart energy to civilian and military missions and smart marine environment monitoring. Therefore, a brief description of their roles in the applicable areas, as well as their current advantages and disadvantages, is necessary.

### 2.1. UAV Network Applications

UAVs are employed in many domains, including agriculture and agricultural water management, where the UAVs are equipped with thermal, multispectral and hyperspectral cameras to acquire data related to crop health via the vegetation index [24]. In this context, low-altitude UAVs offer better spatial and even temporal resolution than satellite imagery [25]. Another use case of UAVs may be found in mineral explorations [26], cryosphere monitoring [27] and landslide monitoring [28], having essential benefits, especially in the context of climate change and hazardous events.

UAVs’ roles can be further extended in other intelligent applications such as real-time road traffic monitoring, where UAV imagery can be used to detect traffic and speed violations [29].

Dynamic UAV-based communication led to the development of the so-called Flying Ad Hoc Network (a multi-UAV network) [30] which has many advantages and applications, as well. Thus, it may imply lower costs than the usual infrastructure, especially when a multi-layer ad hoc network approach is preferred [31]. FANET applications include video transmission enhancement [32], wildfire detection, backup communication in the case of disasters [33] and even water quality monitoring for fish or shrimp farmers [34] Among the advantages of UAV networks are flexibility, acquisition and maintenance cost affordability, survivability and reliability [35]. These capabilities were considered when designing our UAV–USV–WSN network.

### 2.2. USV Network Applications

Water communications began as a response to the necessity of connecting boats and ships to exchange information related to their identity and position. Moreover, there were situations in which the safety of ships and crews was in danger. Nowadays, water communications are critical for use in search and rescue missions, and they were also extended for aquatic environment monitoring applications [36,37] (especially for marine fauna research [38,39] for the localisation of rich fish populations or seismic activity detection [40].

Meanwhile, a new class of unmanned vehicles emerged: unmanned water vehicles, which have roles in hydrographic monitoring, facilitate oceanographic research and maritime or river missions. There are two categories of unmanned water vehicles: underwater vehicles and surface vehicles [41]. Both types of vehicles integrate technologies and intelligent devices such as sensors and wireless networks, thus becoming critical components for the IoT landscape. Compared to human-crewed ships and boats, unmanned vehicles can be often used in missions that imply the inspection of rugged areas.

### 2.3. Architectures for Unmanned Vehicles Networks (UVNs)

The communication between UVs may be classified as follows:

1. UV communication based on a shore control centre. This is a centralised architecture where direct communication between vehicles is not possible. Moreover, due to the position of the surface vehicles and due to the specific characteristics of the regions where they are deployed, i.e., rich vegetation, as in deltas, this configuration may not be recommended because the entire architecture is dependent on the ground control centre. If the ground control centre communication fails or is not in Line-of-Sight (LoS) conditions, the entire network will be compromised.
2. UV communication based on cellular networks. It eliminates the constraints related to the coverage area and UVs’ mobility. Unfortunately, there are areas where cellular base stations are not deployed. Additionally, there are high costs associated with the deployment of new base stations, and operators are discouraged from installing new equipment. Moreover, customers must subscribe for all the UVs connected to the cellular network.
3. UV communication based on satellite constellations. This solution benefits from global coverage, especially in hard-to-reach regions where a cellular network cannot be deployed. Due to disadvantages such as high latency, high energy consumption, the antenna’s dimensions, subscriptions and contract issues or because both the UV and the shore control centre must be in the LoS of a satellite, this solution may not be a good candidate for UV networks.
4. UV communication based on ad hoc networks. This solution allows for the cooperation between UVs, especially by using mesh topology. It may also allow communications with the devices on the shore, thus resulting in the monitoring missions having a higher success rate. In addition, this topology enables cooperation between nodes, as each node can act as a relay [42]. Unlike the solutions mentioned above, the implementation of this network ensures a large coverage area. In addition, it is decentralised, thus eliminating the dependence of a central node. Yet, decentralised communication may result in challenges such as limited communication bandwidth, delays and failures [43].

### 2.4. UAV and USV Network Enhancements

Park et al. [7] proposed an algorithm for UAV fleet control to create an ad hoc wireless network that services a Wireless Area Network connecting terrestrial devices. They succeeded in improving the network’s throughput based on the control of the position of the drones. Their work aimed to reduce congestion by casting out UAVs. In addition, they evaluated the algorithm in ns-3 on a surface of 500 m × 500 m, and they assumed that the terrain was flat. Instead, our algorithm considered the propagation environment and channels and could be applied on larger surfaces and different terrain profiles, because it considered the terrain elevation.

In [44], a path planning concept based on environmental conditions (wind speed and direction) was proposed. It exploited the possibility to change the angle between wind force and UAV path. Nevertheless, there are other factors which influence UAV path planning, such as payload and speed. Our work, though, was based on communication parameters and not environmental ones.

Chen et al. [45] proposed an algorithm for reconstructing an air-to-ground channel and predicting the signal strength between a single UAV and several ground end users. Through the proposed algorithm, the authors in [45] targetted the optimal UAV-based relay position. Their work did not cover all air-related channels and only employed one UAV. Like our work, they aimed to find the optimum position of the UAV such that it acts as a relay. In comparison with their work, the current paper made use of USVs that can act, in their turn, as access points.

In [46], already known air-to-air channel models were revisited and path loss-related simulations were only performed for two UAVs. The work was a precursor initiative of FANET algorithms development. However, most of the employed models (e.g., Egli, Okumura, SUI) have some limitations related to the height and distance ranges. As compared to [46], in our paper, we used UAV propagation models adapted to real-case scenarios. For example, we used the 3GPP model to highlight the propagation losses regarding communication between ground users and UAVs and between UAVs themselves when they are not in the line-of-sight condition, under a height of 60 m. Moreover, the height and distance ranges were broader and closer to real environments.

Several path planning or formation algorithms were developed for USV networks, but all were based on path optimisation or obstacle avoidance [47,48,49,50,51] in the context of autonomous guidance, ignoring the communication layer.

In [52], a communication technology switching algorithm was proposed for shore-to-sea communication. The authors employed a USV equipped with the following technologies: broadband data, satellite communication, shortwave communication, and BeiDou Navigation Satellite System (BDS). Nevertheless, the proposed method was based on a resource allocation approach. Similar to [52], we proposed the switching of the communication technology based on the scenario, i.e., a long-range communication technology (e.g., LoRa/LoRaWAN) is used for minimum low-throughput connectivity between GW and UVs. In contrast, a short-range, high-throughput technology is used to quickly transfer data between a served WSN and UV or between UVs themselves. As opposed to [52], we used a path-loss-based approach and not a resource-allocation-based one.

When referring to UAV–USV cooperation, it is worth mentioning again that the existing solutions consider the positioning of UAVs or USVs from different perspectives. One of them is related to the landing and transportation of a UAV by a USV. The positioning algorithm employs the distance data acquired from four ultrasonic sensors. Even though UVs are equipped with communication modules, communication-related data are not used to enhance UVs positioning [12]. We based our solution on the cooperation between UAVs and USVs as in [12], but our implementation was dedicated to exploiting the connection between more UAVs and USVs to ensure a communication chain, not the physical landing or the transportation of UAVs by USVs, as proposed by [12]. The other approach relates to the cooperation between UAVs and USVs from the point of view of the communication network. Ma et al. [11] proposed a UAV–UVS architecture that was tested from the point of view of the network capacity. In contrast to [11], our architecture only employed one gateway for all UVs. Ma et al. proposed the use of two different gateways, one dedicated to UAVs and one dedicated to UVSs, thus increasing the complexity of the network. Moreover, their viewpoint was concentrated on the routing protocols and network capacity, not on propagation aspects.

Nevertheless, most of the initiatives found in the literature are focused on path planning algorithms, routing algorithms that consider end-to-end delay and packet error ratios, but few formation algorithms approach the FANET or SANET networks from a path loss perspective. The current paper fills this important gap by considering the impact of the propagation environment on the capability of a network to be deployed on demand. Moreover, it proposes the usage of USVs as access points in environments such as the Danube Delta or those at the shore of the rivers and lakes.

## 3. FSANET–WSN Networks: Proposed Architecture and Features

**Figure 1:**
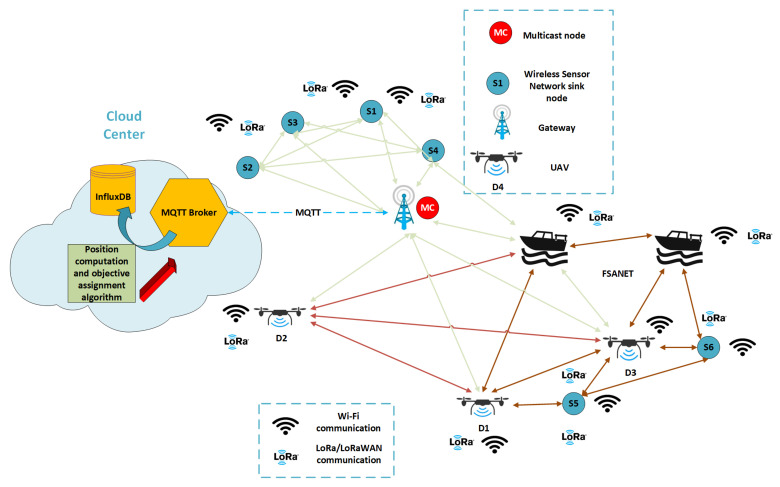
FSANET-WSN (Flying and Surface Ad Hoc NETwork serving Wireless Sensors Networks) architecture.

We proposed the architecture of a Flying and Surface Ad Hoc NETwork serving Wireless Sensors Networks (FSANET-WSNs) on demand (Figure 1).

Each WSN comprises a sink node (or central node) and sensor nodes. The wireless sensors network communicates with the rest of the network through a sink node, as emphasised in Figure 1. There are WSNs capable of communicating directly or/and by multi-hop with the gateway (S1–S4). Concurrently, there are also WSNs that are not in the range of the gateway (S5–S6). This situation appears when the WSNs are deployed in no or weak coverage areas.

The architecture exploits the usage of UVs as an alternative to the costly and often not justified deployment of another fixed wireless infrastructure. UVs can perform several tasks while acting as mobile routers. In addition, given the use cases in which the sampling rate of the sensors nodes exceeds 30 min or even several hours, the wireless infrastructure may be reused and exploited in other locations. The monitoring is also performed on site, as in agriculture, where the sensors are deployed in fixed areas, and the wireless sensors networks are unavoidable. In the context of the Internet of Things, more beneficiaries can share their unmanned vehicles to deploy the on-demand network to cover a more extensive network than a beneficiary alone could have deployed with their own equipment.

To ensure the communication between a WSN and the gateway (GW) through the use of FSANET, the optimum location of the UVs must be determined. To this end, the initial positions of the UVs must be known.

The two communication technologies employed are LoRa/LoRaWAN and Wi-Fi (both in unlicensed spectra). LoRa/LoRaWAN is a low-power wide-area network technology used to transmit infrequent, low-throughput data. LoRa/LoRaWAN is used to send non-critical, non-frequent data inside WSNs and to communicate the Global Positioning System (GPS) coordinates of UVs when they are out of the range of Wi-Fi communication. LoRa refers to the modulation technique used in the physical layer, whereas LoRaWAN also embeds the data protocol to connect devices to the Internet infrastructure. LoRa/LoRaWAN have several limitations, among which include the limited number of messages per day a device can send to the gateway and the far more constrained downlink messages number [53]. From the gateway, the data are sent to the Cloud Center (CC). Here, the positions of the fixed nodes and the last positions of the UVs are stored in an InfluxDB database. In addition to the storage capability of the CC, it coordinates the UVs’ tasks and executes the position optimisation algorithm.

To enable multicast downlink transmission through LoRaWAN—as needed after the CC returns the results of the computation of the optimum positions of the UVs—a multicast node is required. It is in charge of sending a new position to each UV. To reduce the payload size, the Cloud Centre sends back the difference between the computed position and the position forwarded by UV to Cloud Centre to each UV.

Wi-Fi technology is used for a fast transfer between a served WSN and the GW through the FSANET network due to its higher throughput, as FSANET nodes have power constraints related to their operation (for example, their propellers’ functions).

The paper focuses, as mentioned in Section 1, on the development of an algorithm that provides the optimum position for UVs to connect a certain terrestrial WSN to an IoT platform. This section aimed at presenting the framework and the simplified IoT architecture where such an algorithm is necessary. In Section 4, the theoretical background of the algorithm is presented.

## 4. Towards Algorithm Development: Context

Let us assume that we have $N_{rn}=n_{UAV}+n_{USV}$ mobile unmanned vehicles (drones, boats and other surface vehicles) $UV_{1},UV_{2},UV_{3},\ldots ,UV_{i}$, …, $UV_{N}$ that are able to monitor an area $A=A_{1}\cup A_{2}\cup$…$\cup A_{N}$, where $A_{i}\cap A_{j}\neq \emptyset$. Moreover, we have *M* WSNs that communicate with the Cloud Center through a sink node. Some of the sink nodes can be connected directly to the same gateway (noted further as GW) as the Cloud Center, whereas others can be connected at a certain time to the FSANET network, whose nodes become access points for WSNs. The GPS coordinates (latitude, longitude) and the height of the GW and WSNs antennae to the ground are known and stored in the CC database.

The location of each unmanned vehicle is queried on demand by the CC. Thus, the CC triggers the UVs based on a particular message through LoRa communication. In exchange, UVs send their current locations. The CC executes the position optimisation algorithm and sends a set of relative coordinates (offsets) to each UV in range. Specifically, in the case of USVs, the latitude offset (Δϕ) and longitude offset (Δλ) are sent, whereas for aerial vehicles, an extra parameter, altitude offset, is provided (Δa).

The main concern is to connect a certain WSN to the gateway with the help of UVs acting as access points.

UVs are autonomous in the limits of LoRa coverage. When the LoRa communication link is not available, the UVs are programmed to return to a predefined location in the range of the gateway.

**Figure 2:**
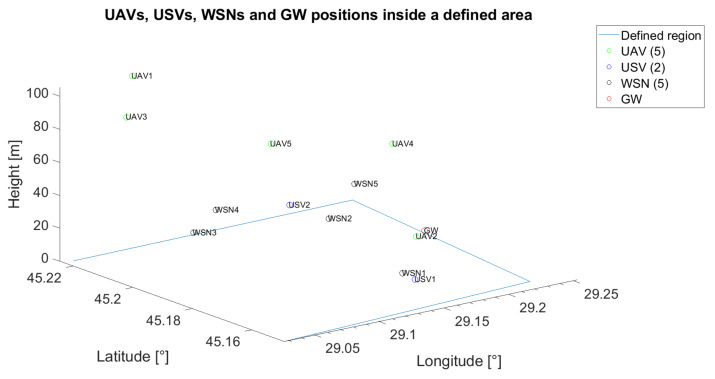
FSANET-WSN network example: $n_{UV}=7$, $n_{UAV}=5$, $n_{USV}=2$, M=5.

The unmanned vehicles can act as relays when the communication between a specific unmanned vehicle or WSN and the gateway is not possible or when the communication between unmanned vehicles themselves cannot be assured through a direct link. In Figure 2, an example of the positioning of five UAVs, two USVs, one GW and five WSNs is emphasised. On the x-axis, the longitude was represented, whereas on the y-axis, the latitude was represented. The z-axis was reserved for antenna height to the ground. For reproducibility, throughout the paper, all the positions of the UVs and of the fixed nodes (GW and WSN) are generated based on pseudorandom number generators with known states.

## 5. Towards Algorithm Development: Propagation Concepts, Path Loss and Communication Graph

When designing a ground wireless network, one of the concerns that arise is the possibility of ensuring the communication link between a transmitter and a receiver at a certain distance and under certain conditions. A more complex scenario resides in the integration of unmanned aerial vehicles into a hybrid wireless network, as an efficient 3D (tri-dimensional) positioning of the UAV must be determined. To correctly plan the positioning of UAVs and USVs, when referring to the quality of the communication, one must describe the parameters involved, the propagation environments and propagation channels and models.

### 5.1. Link Budget

The link budget represents a measure of the performance of the communication link and accounts for the gains and the losses that occur along the entire transmitter–receiver chain [54]. The general Equation (for expressing the link budget is given in Equation (1).

(1) $$P_{rx}\left[dBm\right]=P_{tx}\left[dBm\right]+G_{tx}\left[dB\right]+G_{rx}\left[dB\right]-L_{tx}\left[dB\right]-L_{rx}\left[dB\right]-PL\left[dB\right]$$

where $P_{rx}$ is the received power, $P_{tx}$ represents the transmitted power, $G_{tx}$ is the antenna gain of the transmitter and $G_{rx}$ corresponds to the antenna gain of the receiver. $L_{tx}$ and $L_{rx}$ delineate the losses occurring on the passive network (e.g., connectors, cables) [55]. Finally, PL (path loss) accounts for the losses that occur across the propagation environment, being estimated based on a propagation model specific to a specific scenario and environment in the absence of fading.

In a real environment, for the communication between nodes that change their position (as those proposed in the paper), omnidirectional antennae (equally radiating in all horizontal directions) are desired. Usually, they have a gain of 2.15 dBi.

Nevertheless, further, we assume that the antennae are isotropic with $G_{tx}=G_{rx}=0$ dBi and that the transmitter and receiver losses through the connectors and other components are $L_{tx}=L_{rx}=0$ dB. Therefore, the link budget can be computed using Equation (2).

(2) $$P_{rx}\left[dBm\right]=P_{tx}\left[dBm\right]-PL\left[dB\right]$$

Further, we introduce a new parameter, the fade margin (μ), which is the additional transmitter power necessary in the presence of fading such that to increase the received signal power until the same level of the received signal power that would have been obtained in the absence of fading. The relation between the transmitter power in the absence of fading $P_{tx}$, the transmitter power in the presence of fading $P_{tx_{f}}$ and the fade margin, μ, is given in Equation (3).

(3) $$\mu \left[dB\right]=P_{tx}\left[dBm\right]-P_{tx_{f}}\left[dBm\right]$$

For the communication link to be ensured, the received power must be higher than or equal to the sensitivity of the receiver, $S_{rx}$ (Equation (4)).

(4) $$P_{tx_{f}}\left[dBm\right]-PL\left[dB\right]\geq S_{rx}\left[dBm\right]$$

The difference between the transmitted power, the receiver sensitivity and the fade margin (Equation (5)) is referred to as the link budget and is denoted by LB.

(5) $$LB\left[dB\right]=P_{tx}\left[dBm\right]-S_{rx}\left[dBm\right]-\mu \left[dB\right]\geq PL\left[dB\right]$$

In Table 1, several technologies’ and parameters’ values, retrieved from the datasheets of available communication modules on the market, are highlighted. To emphasise the suitability of a transceiver for a use case, the data rate (DR) is also given. When computing the link budget, we considered a fade margin of 20 dB as being sufficient for critical telemetry data transmissions [56].

Concerning LoRa communication, in Europe, both 868 MHz and 433 MHz ISM (Industrial, Scientific and Medical) bands can be used. We chose a transceiver operating on 868 MHz based on the comparison between the characteristics of each frequency plan (Table 2).

The allowed transmitter (TX) power in the 868 MHz band is higher than in the 433 MHz band. In addition, the dimension of the antenna doubles when using the 433 MHz band. It is also known that the 868 MHz band is less congested than 433 MHz. The packet reception is more stable in the 868 MHz band, whereas the penetration capability is reduced compared to the 433 MHz band. The penetration capability is useful, though, in indoor applications, which is not the case for the applications presented throughout the paper. Another aspect regarding 868 MHz frequency resides in its better overall performances at the edge of the coverage area, as it is useful for a less dense network operating at a higher distance from the gateway, as proposed in the paper.

### 5.2. Propagation Environment and Channels

The environment has a significant impact on the propagation models because physical aspects, such as the existence of buildings and their heights, the terrain and the level of vegetation, impact propagation. There are six types of propagation environments: (1) free space, (2) rural, (3) suburban, (4) urban, (5) dense-urban and (6) high-dense urban. In our paper, we only approach rural and free space situations, as they are more specific to isolated areas.

Moreover, from the propagation channels available in the literature, we extracted and adapted the following classification to our use cases:

- Aerial and terrestrial channels
  - Air-to-ground channel (A2G)
    Propagation channel for communication between aerial vehicles and ground nodes, when aerial vehicles transmit to ground devices.
  - Ground-to-ground channel (G2G)
    Propagation channel for communication between ground devices.
  Ground-to-air channel (G2A)
    Propagation channel for communication between aerial vehicles and ground nodes, when ground nodes transmit to aerial vehicles.
  - Air-to-air channel (A2A)
    Propagation channel for the communication between aerial vehicles.
  It is worth mentioning that air-to-ground channel characteristics are also reflected in the ground-to-air channel features [62].
- Water channels
  - Ship-to-shore channel (S2S)
    Propagation channel for communication between vessels on sea and devices placed onshore.
  - Over-water channel (OW)
    Propagation channel for communication between ships on sea, rivers or lakes.
  - Air (UAV)-to-ship channel (A2S) [63]
    Propagation channel for communication between ships on water or ports and aerial vehicles.

The following sections describe the effects of the considered channels by large-scale models, that is, from the point of view of path loss and the propagation environment [64].

### 5.3. Air-to-Ground Channel (A2G)

The air-to-ground channel oversees the ability of an unmanned aerial vehicle to provide a communication link for the ground nodes. Due to the high altitude they can reach, the channel conditions for UAV will be improved, as compared to standard terrestrial base stations [22]. Nevertheless, when the communication between a UAV and a mobile base station is desired, one must consider the fact that the base station antenna is down tilted, such that the ground coverage is optimised, to the detriment of UAV coverage [65]. According to International Telecommunication Union (ITU) studies [66], for urban/suburban regions, buildings and vegetation impact in a more significant measure the quality of communication than multipath reflections. Consequently, for our purpose, multipath reflections are not taken into consideration, supposing the deployment of the proposed solution in a high vegetation index area.

Furthermore, for the simplicity of the model, given the low node density and the small probability that other similar networks are deployed in the area, we do not take into account the interfering nodes.

#### 5.3.1. Path Loss Modelling

Further, one must consider the path loss model for air-to-ground channels. In the literature, a consistent number of air-to-ground PL models were proposed. In [67], 16 path loss models are reviewed, while in [68], a comparison of the most significant air-to-ground channel models is presented in terms of proposed scenarios, advantages and drawbacks.

#### 5.3.2. 3GPP Models

Reference models of the path loss for aerial vehicles are provided in 3GPP TR 36.777 (Table B-2) [69]. Even though they are too complicated for use in performing theoretical analysis or for the offline optimisation of UAV trajectories, they are very useful for numerical simulations [68]. The models target three scenarios: Rural Macro-cell with the model Rural Macro (RMa-AV) in LoS conditions, Urban Macro-cell with the model Urban Macro (UMa-AV) in NLoS conditions and Urban Micro-cell with its corresponding model, Urban Micro (UMi-AV). The specific usages of each scenario are depicted in Table 3. In this paper, only the Rural Macro-cell scenario is envisioned, considering a use case for the Danube Delta or another isolated area where the rural environment prevails.

### 5.4. Air-to-Air Channel

When no highly directive antennae are employed, the air-to-air channel is affected by multiple ground reflections. Additionally, due to the flying speed, UAV–UAV communication experiences considerable Doppler shifts. For our approach, though, we assumed that the UAVs were stationary at the moments when they facilitated the communication between the desired WSNs and the other nodes. In addition, the elevation angle was ignored for the air-to-air channel [62]. Consequently, we propose the employment of the free space propagation model.

#### Path Loss Modelling: Free Space Model

The simplest model consists of the free space path loss (FSPL) approximation given by Equation (6).

(6) $$\begin{array}{c} FSPL=20\;\cdot \;\log_{10}d+20\;\cdot \;\log_{10}\left(f\right)+20\log_{10}\left(\frac{4\;\cdot \;\pi }{c}\right) \end{array}$$

where *d* is the geodesic or ground distance between the UAV and ground node expressed in km, *f* is the operating frequency in MHz and *c* is the wave propagation speed ($c=3\times 10^{8}$ m/s). The free space path loss model can only be applied when two conditions are fulfilled:

1. There are no obstacles in the line of sight path between the UAV and ground user;
2. There are no objects in the first Fresnel zone [72].

In addition, the free space scenario implies the absence of any reflecting surface, and it does not consider the Earth reflections.

The free space propagation scenario is valid until the radio signals transmitted by the UAV reach one of the following environments: rural, suburban, urban, dense urban, high-dense urban [73].

### 5.5. Air-to-Ship Channel (A2S)

Usually, air-to-ship channels are characterised by the LoS path and reflection path [74]. The latter is due to the reflection of radio waves on the water surface. In addition, due to reflections, there is a possibility that deep nulls appear when the signals compose destructively [75]. In the literature, according to our research, there are many empirical models proposed for an air-to-sea channel [63,76], which is a particular example of a water channel, and especially for ultra high-frequency (UHF) and very high-frequency (VHF) communications where large antennae are used, but very few deterministic models or models adapted to other water environments such as big rivers or lakes or to other communication technologies exist. Moreover, few models can be applied to different communication frequencies. A couple of those models are the two-ray model and the three-ray model. To consider the LoS and reflected paths, the two-ray model given by Equation (7) can be used [63,77].

(7) $$PL_{2-ray}=20\;\cdot \;log_{10}\left(\frac{4\pi fd}{c}\right)-10\;\cdot \;log_{10}{\left[2\sin\left(\frac{2h_{t}h_{r}f}{cd}\right)\right]}^{2}$$

Based on the model, another parameter, the critical distance, can be computed. The critical distance (Equation (8)) reveals the distance threshold after which the path loss increases with $d^{4}$.

(8) $$d_{c}=\frac{4h_{t}h_{r}c}{f}$$

By also considering the evaporation due to the existence of a duct layer of height, $h_{d}$, the three-ray model is obtained (Equation (9)).

(9) $$PL_{3-ray}=20\;\cdot \;log_{10}\left(\frac{4\pi fd}{c}\right)-10\;\cdot \;log_{10}{\left[2(1+\Delta )\right]}^{2}$$

Equation (10) represents the relationship used to compute Δ.

(10) $$\Delta =2\sin\left(\frac{2\pi h_{t}h_{r}f}{cd}\right)\sin\left[\frac{2\pi \left(h_{d}-h_{t}\right)\left(h_{d}-h_{r}\right)f}{cd}\right]$$

Table 4 reviews the parameters mentioned in two-ray and three-ray path loss models.

Based on Equations (7) and (8), we performed the following simulations for the transceivers in Table 1.

**Figure 3:**
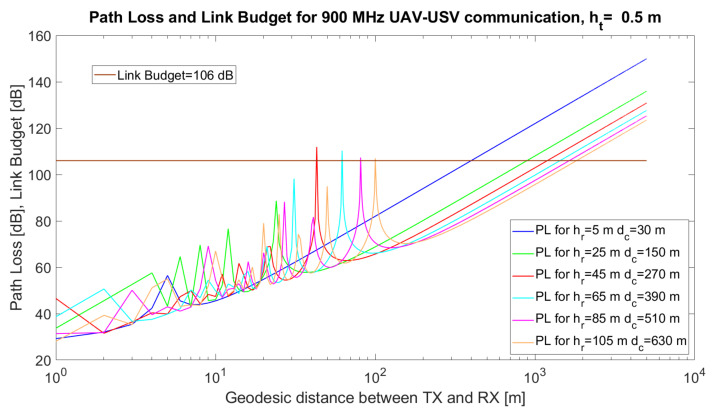
Path loss, link budget and critical distance for UAV–USV link, when USV antenna height is $h_{t}$ = 0.5 m and the solution in [57] is used.

In Figure 3, the simulated path loss, link budget and critical distance for a UAV–USV link are emphasised. The simulations were performed using MATLAB 2019b for communication between a USV and a UAV. The simulation parameters and their values are given in Table 5.

**Figure 4:**
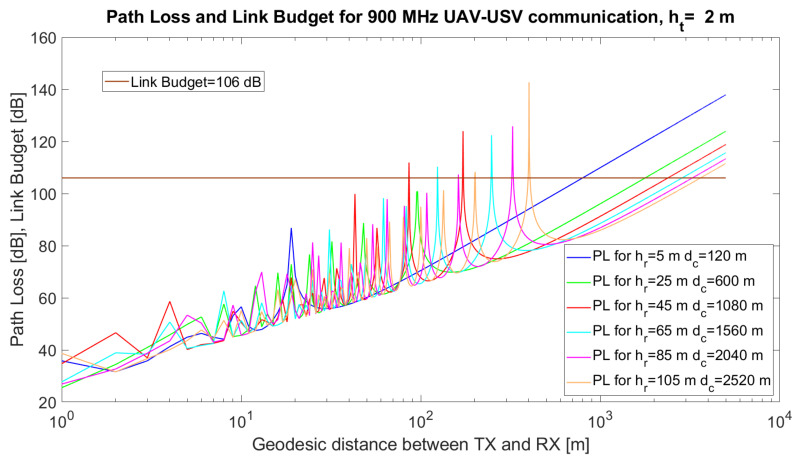
Path loss, link budget and critical distance for UAV–USV link, when USV antenna height is $h_{t}$ = 2 m and the solution in [57] is used.

**Figure 5:**
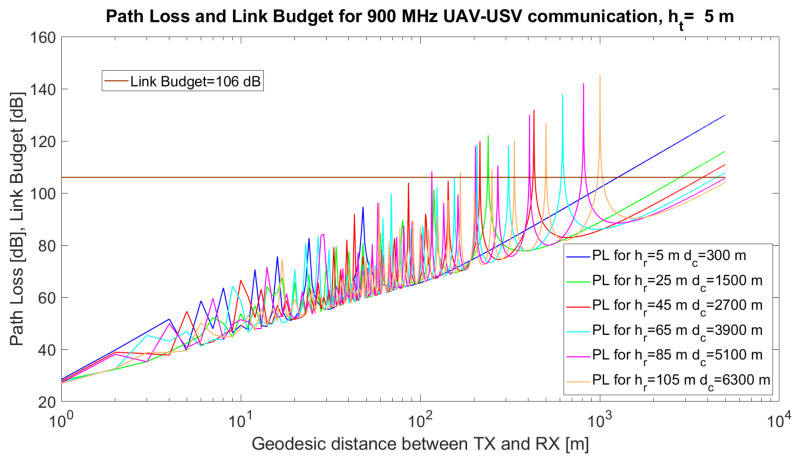
Path loss, link budget and critical distance for UAV–USV link, when USV antenna height is $h_{t}$ = 5 m and the solution in [57] is used.

The technical solution implies the use of the communication modules in [57]. The height of the USV antenna varied from $h_{t}$ = 0.5 m to $h_{t}$ = 5 m when higher USVs were implied in the simulations. The height of the UAV varied from 5 m to 120 m (the maximum allowed altitude to comply with the UAV regulations). The results are given in Figure 3, Figure 4 and Figure 5.

It can also be observed from the simulations that, at certain distances, *d*, the UAV–USV communication path loss has maxima, due to the destructive composition of the radio signals. Mathematically, it can be proven that the argument of the sinusoidal function in the two-ray model, $\frac{2h_{t}h_{r}f}{cd}$, reaches values close to π for certain combinations ($d,h_{t},h_{r}$). Thus, the value of the sinus function tends towards 0, while the logarithm tends towards −∞, Consequently, the overall path loss would tend towards *∞*.

When $h_{t}=0.5$ m (Figure 3) or $h_{t}=2$ m (Figure 4), there are several maxima that exceed the link budget, at specific values of $h_{r}$, but in the case of higher values of $h_{t}$ (Figure 5) the number of maxima exceeding the link budget increases further. From the simulations, it can be observed that a very important task of the algorithm resides in the estimation of the optimum tuple $\left(d,h_{t},h_{r}\right)$ which will be translated to tuple $\left(\phi_{i},\lambda_{i},\phi_{j},\lambda_{j},\epsilon_{i},\epsilon_{j}\right)$ in order to minimise the path loss and avoid the occurrence of signal loss due to the destructive composition of the signals.

Different recommendations may arise from the simulations. For $h_{t}$ = 5 m, the results are given in Table 6.

Nevertheless, it would be challenging to treat all the scenarios that may arise and find an optimum (d, $h_{t}$, $h_{r}$) tuple following a non-automate or a traditional mathematical model, as one has to ensure many such communication links, not only one.

### 5.6. Overwater Channels (OW) and Ship-to-Shore Channels

**Figure 6:**
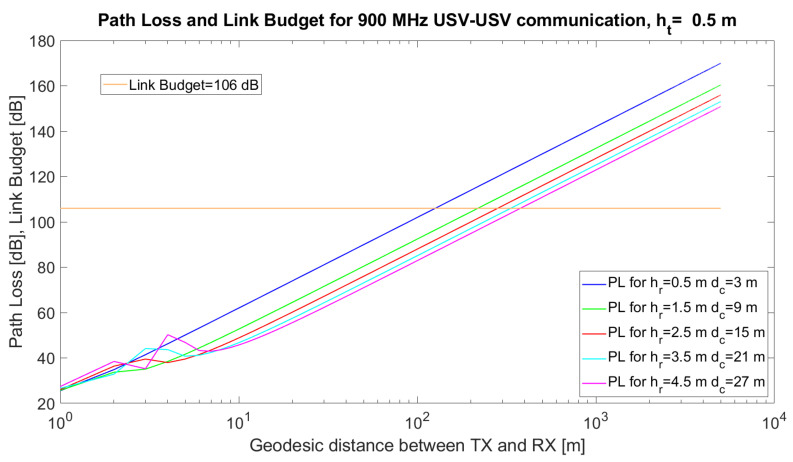
Path loss, link budget and critical distance for USV–USV link, when USV antenna height is $h_{t}$ = 0.5 m and the solution in [57] is used.

Overwater channels or ship-to-ship and ship-to-shore channels are more affected by physical phenomena than the air-to-ship channel, since both transmitter and receiver antennae are close to the water surface. If we would apply the same two-ray model (Equation (7)) and the same technical solution (transceiver in [57] for small USVs, the coverage distance would not be more than 120 m when both heights of the antennae only reach 0.5 m. The coverage reaches 373 m when it is possible to place the antenna in a higher position (4.5 m) for one of the USVs (Figure 6).

The most problematic channel is related to the water environment. Consequently, we want to prove that the path loss characterising UAV–USV, USV–USV or USV–GW/WSN communications is minimum in terms of antenna height but zero in terms of distance. To this end, we handle Equation (7), and we suppose $h_{r}$ is the height of the USV antenna, whereas $h_{t}$ is the height of the UAV antenna. Because the USV cannot move vertically (on the z-axis), we state that $h_{r}$ is constant, whereas the UAVs can increase or decrease their altitude and consequently, $h_{t}$ is variable. In addition, the distance between UV increases or decreases as well. To conclude, both $h_{t}$ and *d* are variables with respect to $PL_{2-ray}$. We cannot find a minimum PL in terms of the distance because Equation (11) does not have solutions for d>0.

(11) $$\frac{\partial PL_{2-ray}}{\partial d}=0$$

**Proof.**  (12) $$\frac{\partial PL_{2-ray}}{\partial d}=\frac{2}{d\ln10}\left(1+\frac{\beta }{d}\cot\frac{2\beta }{d}\right)=0$$

where $\beta =\frac{h_{t}h_{r}f}{cd}>0$. By denoting $\frac{\beta }{d}=\alpha >0$, we will solve Equation

(13) 1+αcotα=0

with the unique negative solution α=−227.769 rad which contradicts the afore-mentioned condition for α.    □

Instead, we can find a minimum of PL with respect to $h_{t}$ by solving Equation (14).

(14) $$\frac{\partial PL_{2-ray}}{\partial h_{t}}=0$$

(15) $$\frac{\partial PL_{2-ray}}{\partial h_{t}}=-10\;\cdot \;\frac{\partial \left[log_{10}{\left[2\sin\left(\frac{2h_{t}h_{r}f}{cd}\right)\right]}^{2}\right]}{\partial h_{t}}$$

(16) $$\frac{\partial PL_{2-ray}}{\partial h_{t}}=-\frac{40}{\ln10}\;\cdot \;\frac{h_{r}f}{cd}\cot\left(\frac{h_{t}h_{r}f}{cd}\right)=0$$

for

(17) $$h_{t}=\frac{(2k+1)\pi cd}{4h_{r}f}$$

We replace Equation (17) in Equation (7) and we obtain Equation (18) and (19).

(18) $$PL_{2-ray}\left(d\right)=20\;\cdot \;log_{10}\left(\frac{4\pi fd}{c}\right)-10\;\cdot \;log_{10}{\left[2\sin\left(\frac{(2k+1)\pi }{2}\right)\right]}^{2}$$

(19) $$PL_{2-ray}\left(d\right)=20\;\cdot \;log_{10}\left(\frac{4\pi fd}{c}\right)-10\;\cdot \;log_{10}\left(4\right)$$

Further, we must compute the distance, *d*, for which $PL_{2-ray}\left(d\right)$ is lower or equal than the link budget, *LB*, expressed in dB.

(20) $$PL_{2-ray}\left(d\right)=20\;\cdot \;log_{10}\left(\frac{4\pi fd}{c}\right)-20\;\cdot \;log_{10}\left(2\right)\leq LB\left[dB\right]$$

By solving Equation (20), we found that the distance, *d*, must follow Equation (21):

(21) $$d\leq \frac{c\;\cdot \;10^{\frac{LB+20log_{10}\left(2\right)}{20}}}{4\pi f}$$

We introduce the following hypothesis: all USVs have the same antennae height. Consequently, in the case of communication between two USVs, given the hypothesis, $h_{t}=h_{r}$ and Equation (17) would lead to Equation (22).

(22) $$d=\frac{4fh_{t}^{2}}{(2k+1)\pi c}$$

### 5.7. Path Loss Graph. Communication Graph

Further, we introduce the concept of a path loss graph, which is based on the path loss and link budget parameters. Let $G=(V\left(G\right),E\left(G\right),\psi_{G})$ be the path loss graph, a finite, non-trivial, directed graph where

$$V\left(G\right)=\left(\nu_{1},\nu_{2},\ldots ,\nu_{N}\right)$$

$$E\left(G\right)=\left(e_{1},e_{2},\ldots ,e_{N^{2}}\right)$$

are the network nodes (UVs, GW, WSNs) and the communication links between every two such nodes.

The computation of each weight, *w* (Equation (23), involves (1) the path loss computed between every two nodes of the network following the specific propagation model and (2) the link budget available for the technical solution used at a particular time, LB (Table 1).

(23) $$w_{i,j}=\frac{PL_{v_{i},v_{j}}}{LB}+\zeta$$

with $w_{i,j}\in \left[\zeta ,\infty \right)$, where ζ is infinitesimal. The presence of infinitesimal ζ is required for implementation purposes, as $w_{i,j}\neq 0$ even when $PL_{v_{i},v_{j}}=0$. The communication link is said to be ensured if $PL_{v_{i},v_{j}}<LB$ and, consequently, $w_{i,j}>1+\zeta$

Further, we introduce the concept of a communication graph, which is a subgraph *H* of the path loss graph G, where all the edges with weights higher than 1+ζ were removed, H⊆G, $E\left(H\right)=E(G,w_{i,j}\leq 1+\zeta$). Therefore, the communication graph only comprises the edges corresponding to the nodes that communicate between each other (PL≤LB). We must recall that graph representation does not give visual information about the distances or the position in the space of the nodes.

With all these concepts discussed, Table 7 highlights the environment, channels, scenarios and corresponding path loss models employed throughout the rest of the paper.

**Figure 7:**
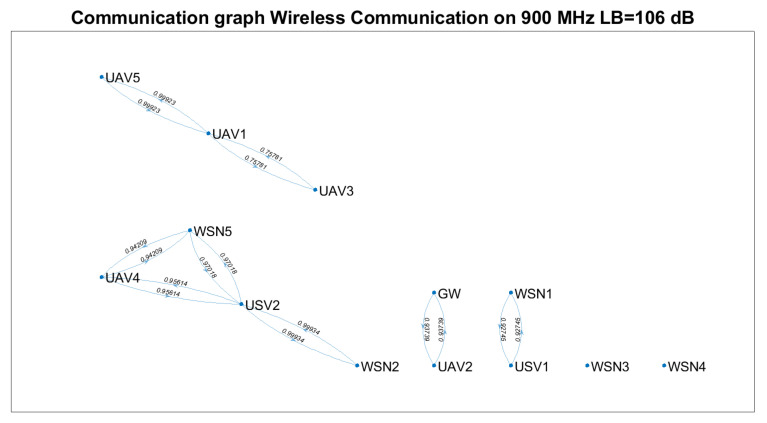
Communication graphs for the technical solutions in [57].

**Figure 8:**
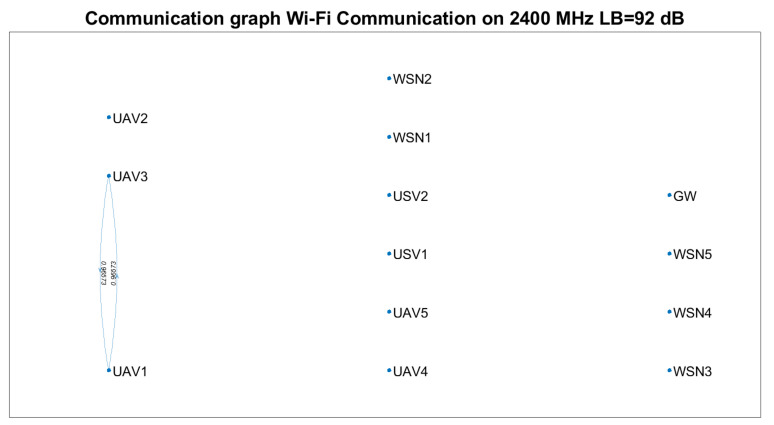
Communication graphs for the technical solutions in [58].

**Figure 9:**
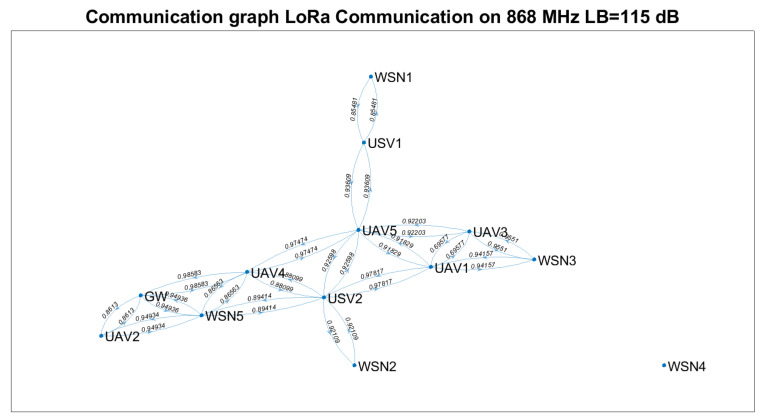
Communication graphs for the technical solutions in [59].

Based on Equation (23) and technical solutions in [57,58,59], the communication graph for each solution was determined (Figure 7, Figure 8 and Figure 9). The positioning of the nodes in the 3D space is kept as in Figure 2 for all four technical solutions, and the height threshold for which we applied the FSPL propagation model for the A2A channel is h=60 m. The graph vertices are the network’s nodes. The blue lines are the edges of the graph. If there is a communication link between two nodes, then such an edge (blue line) will connect them. The existence of two edges between each of the two nodes represents a bidirectional link. We mention that the position of the graph’s vertices or the length of the edges are not related to the position of the nodes in 3D space. Instead, the layout of the graph was automatically chosen by the simulation tool based on the graph’s size and structure. The figures on each edge represent the edges’ weight computed based on Equation (23).

Based on Figure 7, Figure 8 and Figure 9, we notice that the scenarios dramatically change as the communication technology cannot ensure the communication link. Thus, we highlight the importance of a positioning algorithm that adapts to the performances of the communication technology employed at a certain time.

Table 8 summarises the results obtained in terms of several key performance indicators (KPI), such as: the number of connected nodes, number of edges, minimum and maximum weights. We remark that the communication link cannot be ensured between the gateway and each WSN for any of the technical solutions above.

Based on Table 7 and Table 8 and Figure 7, Figure 8 and Figure 9, the following assumptions must be considered:

- Ground-to-ground communication between WSNs and GW is not always possible, and UAVs and USVs act as intermediate gateways.
- The communication links between a gateway and WSN must be adjusted so that none of the intermediate links are fostered to the injury of others (e.g., $w_{min}=0.75781$ versus $w_{min}=0.99934$ ).

Supposing that a WSN must send data to the gateway at a specific moment, the proposed algorithm should be able to find which vehicles must be selected and their positions such that the communication link is possible.

Based on LoRa communication, the communication links can be ensured for a significant percentage of the nodes. Nevertheless, considering the limitations of LoRa technology (low throughput, high latencies and packet delivery time) and the restrictions imposed for UAVs (limited battery life, weather conditions, tear), one should only rely on such a network for limited scenarios. Thus, we envision the provisional use of a LoRa-based ad hoc network to send the locations of the nodes to the gateway at the trigger time and the optimum position data determined based on the proposed algorithm from the gateway to the nodes, such that a higher throughput, lower latency on-demand Wi-Fi network is deployed.

## 6. Proposed Algorithm: Communication Optimisation Integration

### 6.1. Optimum UV Placement and Great Circle Path

The problem of finding the optimum location of a mobile node is an NP-hard (Non-deterministic Polynomial) problem that is very demanding from the point of view of computational resources.

The great circle path or orthodrome, which is the shortest path between two points on a circle (in our case, the gateway and the served WSN), is widely used in navigation, but for the first time, according to our knowledge, we propose the use of this concept as a method to reduce the number of possible locations a UV can have, and thus, the complexity of the NP-hard problem. Consequently, before proposing the placement optimisation algorithm, one requires the presentation of great circle method and its corresponding algorithm.

**Figure 10:**
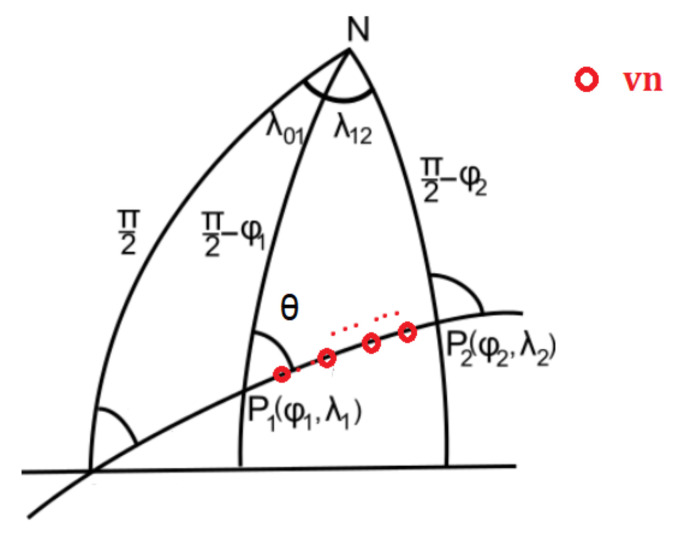
Great circle path between GW ($P_{1}$) and WSN ($P_{2}$) adapted from [78].

We assume there are $n_{vn}$ virtual nodes with the same propagation characteristics that could ensure communication between the gateway and served WSN. The virtual nodes are equally spaced on the great circle path (Figure 10) that links the gateway found on point P1 ($\phi_{1},\lambda_{1}$) and the served WSN placed on P2 ($\phi_{2},\lambda_{2}$).

To ensure their positioning on the great circle path, we computed the forward azimuth (θ) between the two terminal nodes (P1 and P2) computed following Equation (24) [79].

(24) $$\begin{array}{c} \theta {[}^{\circ }]=\left(\frac{180\arctan(y,x)}{\pi }+360\right)\;\mathrm{mod}\;360\\ y=\sin\left(\lambda_{2}-\lambda_{1}\right)\cos\left(\phi_{2}\right)\\ x=\cos\left(\phi_{1}\right)\sin\left(\phi_{2}\right)-\sin\left(\phi_{1}\right)\cos\left(\phi_{2}\right)\cos\left(\lambda_{2}-\lambda_{1}\right) \end{array}$$

The imposed distance, $d_{vn}$, between two virtual nodes is computed based on Equation (25).

(25) $$d_{vn}=\frac{d}{N_{vn}+1}$$

Based on an imposed distance between virtual nodes ($d_{vn}$) and computed forward azimuth, we can compute the location of the virtual nodes points (Equation (26) and (27) [79]).

(26) $$\phi_{i+1}=\arcsin\left(\sin\phi_{i}\cos\left(\frac{d_{vn}}{R}\right)+\cos\phi_{i}\sin\left(\frac{d_{vn}}{R}\right)\cos\theta \right)$$

(27) $$\lambda_{i+1}=\lambda_{i}+\arctan\left(\sin\theta \sin\left(\frac{d_{vn}}{R}\right)\cos\phi_{i},\cos\left(\frac{d_{vn}}{R}\right)-\sin\phi_{i}\sin\phi_{i+1}\right)$$

where *R* is the Earth radius (R=6.371 km).

Further, we propose Algorithm 1 to determine the closest real nodes to virtual nodes.

Algorithm 1 is used to determine the real UV nodes (rn) that are closer to the virtual nodes (vn) such that one minimises the distance travelled by real nodes to the positions of the virtual nodes.
**Algorithm 1** Closest real UV node algorithm**Input:**$N_{vn}$, $N_{rn}=n_{UAV}+n_{USV}$; GPS coordinates**Output:** CN (closest real nodes vector), CD (closest nodes distances with respect to virtual nodes)1:Determine the distances between each virtual node $vn_{i}$ and each real node $rn_{j}$, $D=\left[\begin{array}{c} d_{i,j} \end{array}\right]$$i\leq N_{vn}$, $j\leq N_{rn}$2:Reshape *D* matrix as $BD_{1\times (N_{vn}\;\cdot \;N_{rn})}$3:Sort BD in ascending order as $BD_{1}$4:Find vector bd (index positions of $BD_{1}$ in BD)5:Initialize closest real nodes vector $CN_{k}=0\forall k\leq N_{vn}$6:Initialize visited real nodes vector AT=∅7:**for** i←1to$N_{rn}$**do**8:   $c\leftarrow bd_{i}/N_{vn}$9:   $r\leftarrow bd_{i}\%N_{vn}$10:  **if** r>0 **then**11:     n←c+112:  **else**13:     $r\leftarrow N_{rn}$14:     n←c15:  **end if**16:  **if** $CN_{n}=0\;\&\;r\notin AT$ **then**17:     $CN_{n}\leftarrow r$18:     AT←AT∪{r}19:     $CD_{n}\leftarrow d_{n,r}$20:  **end if**21:**end for**

Because of the devices’ heterogeneity and the influence of distance and height between nodes, the virtual nodes’ initial placement will not ensure end–end communication. Consequently, based on the communication graph and optimisation algorithm (Algorithm 2), the optimum solution in terms of distance (latitude, longitude) and height are found.

Thus, we started with a hypothetical placement solution assuming that real nodes are placed in the same positions as homologous closest virtual nodes. Algorithm 1 was evaluated, and the results are given in Table 9.

We observe that for $n_{UV}=n_{UAV}+n_{USV}\leq$ 20, neither the number of UVs nor the distance between WSN and GW does not impact the execution time significantly. The algorithm was also tested for $n_{UV}$ = 52, where the execution time increased by 0.0132 s as compared to the scenario when $n_{UV}=20$, for the same distance of 5 km. When $n_{UV}=100$, the execution time increases 2.33 times with respect to $n_{UV}=52$ and 3.68 times with respect to $n_{UV}=10$. Consequently, we notice that the distance between the gateway and WSN has a lower impact on the time performance than the number of UVs employed in the simulations.

The proposed great circle approach together with Algorithm 1 is useful for reducing the search space for the optimal position, determined further using Algorithm 2. If instead of Algorithm 2 proposed further, one would use backtracking algorithm—suitable for solutions to such NP-hard problems, the complexity would increase to n×k, where n is the number of UVs and k is the number of different positions each of them can have in the entire space.

### 6.2. Position Optimisation Algorithm

The goal of the proposed placement optimisation algorithm consists of minimising the cost function $f_{0}\left(\Delta ,\chi \right)$ (Equation (28)).

(28) $$\begin{array}{ccc} \; & \underset{\Delta_{j},\chi_{j}}{\mathrm{minimise}}\; & f_{0}\left(\Delta ,\chi \right)=\sum_{n=1}^{vn}a_{j}\Delta_{j}+b_{j}\chi_{j},\\ \; & \mathrm{subject}\;\mathrm{to}\;w_{j,j+1}<1+\zeta & \; \end{array}$$

where $j\in \{1,\ldots ,N_{vn}\}$, $\Delta =[\Delta_{j}]$ is the distance travelled by $rn_{j}$ and $\chi =\left[\chi_{j}\right]=|\left[h_{f}\right]-\left[h_{i}\right]|$ is the height offset, with $h_{f}$ and $h_{i}$ being the final and the initial height of $rn_{j}$, respectively (only the UAV modifies its height). $a_{j}$ and $b_{j}$ are the distance weight and height weight, respectively. The optimisation problem implies a linear optimisation function with non-linear constraints, representing a non-linear programming problem (NLP).

Based on the characteristics of the propagation channels, the heights of the antennae and propagation loss, Algorithm 2 computes the latitude, longitude and elevation of the optimum positions. Therefore, the communication link is assured with minimum cost in UVs horizontal and/or vertical displacement.

The algorithm uses an adaptive distance step, Δd, to increase its performance and decrease its execution time when PL>LB and a fixed small step for ($PL<LB-\Delta_{LB}$) to avoid the significant changes of PL, which would especially afflict the communication between USV and UAV or between USVs. The adaptive step is computed based on Equation (29).

(29) $$\Delta d=\rho d_{0}{}_{i,j}$$

where ρ represents the coefficient for adaptive distance and $d_{0}{}_{i,j}$ is the distance between node *i* and node *j* at the previous iteration.


**Algorithm 2** Path loss-based placement optimisation algorithm**Input:**$n_{UAV}$, $n_{USV}$, $N_{vn}$, $N_{rn}$ GPS coordinates; served WSN; $h_{th}$ {Maximum allowed UAVs altitude}; $h_{FSPL}$ {Minimum UAVs altitude to apply FSPL model};δh {Height step} *LB* {Link Budget}, δd {Distance step for $PL<LB-\Delta_{LB}$}, Δd {Adaptive distance step for PL>LB}, ρ {Coefficient for adaptive distance step Δd}.**Output:** Optimum latitude $\phi_{opt,n_{i}}$, longitude $\lambda_{opt,n_{i}}$, elevation $\epsilon_{opt,n_{i}}$ for each UV
1:Initialize $d_{th}$ {Maximum allowed distance offset}2:Compute initial 2D and 3D distances between each node, $d_{i,j}^{2D}$, $d_{i,j}^{3D}$3:Assign path loss model based on the IDs and position parameters of each two nodes (A-G, A-W, A-A, W-G, W-W, G-G)4:Compute initial path loss $PL(n_{i},n_{j})$5:Generate path loss graph G(PL)6:

$cr\leftarrow n_{GW}$

7:

k←0

8:**while** $k\leq N_{vn}\;\&\;P=\emptyset$**do**9:    ek←0 {Height exponent}10:   t←011:   next←CN[k]12:   **if** $next>n_{UAV}$ **then**13:      changeh←0 {USVs cannot change antenna height}14:   **else**15:      changeh←116:   **end if**17:   im←118:____________________________________________________________________________________________________
    **Adjust distance such that**
LB−ΔLB<PL<LB19:____________________________________________________________________________________________________
20:   cr←nodGW21:   **while** $PL_{cr,next}<LB-\Delta LB\;\&\;im=1$ **do**22:      Store previous state ($\phi_{0}\leftarrow \phi_{next}$, $\lambda_{0}\leftarrow \lambda_{next}$, $w_{0}\leftarrow w$, $d_{0}^{2D}\leftarrow d^{2D}$, $d_{0}^{3D}\leftarrow d^{3D}$)23:      $\theta \leftarrow ∠(tx_{cr}.tx_{next})$24:      $\phi_{next}\leftarrow F\left(\phi_{0},\delta d,\theta \right)$ {new latitude}25:      $\lambda_{next}\leftarrow G\left(\lambda_{0},\delta d,\theta \right)$ {new longitude}26:      Compute $PL_{cr,next}$27:      **if** $w_{cr,next}<1+zeta$ **then**28:          **if** $PL_{cr,next}\geq PL_{0_{cr,next}}$ **then**29:              imp←130:              Compute $PL_{j,next}$$1\leq j\leq N_{rn}$31:          **else**32:              imp←033:              Restore previous state: $\phi_{next}\leftarrow \phi_{0}$ {old latitude}, $\lambda_{next}\leftarrow \lambda_{0}$ {old longitude}, $PL\leftarrow PL_{0}$, $d^{2D}\leftarrow d_{0}^{2D}$, $d^{3D}\leftarrow d_{0}^{3D}$)34:          **end if**35:       **else**36:          Repeat Steps 32–3337:        **end if**38:    **end while**39:    imp←140:____________________________________________________________________________________________________
    **Height optimisation**41:____________________________________________________________________________________________________
42: 43:    **if** changeh=1 **then**44:        **while** $\left(imp=1\right)\;\&\;\left(w_{cr,next}>1+\zeta \right))$ **do**45:            $h_{next}^{0}\leftarrow h_{next}$46:            $h_{next}\leftarrow h_{next}^{0}+\left({\left(-1\right)}^{ek}\right)*\delta h$47:            **if** $h_{next}<h_{th}$ **then**48:                Store previous state ($w_{0}\leftarrow w$, $d_{0}^{2D}\leftarrow d^{2D}$, $d_{0}^{3D}\leftarrow d^{3D},h_{next}^{0}$)49:                $d^{3D}=H\left(d^{2D},h_{next}\right)$50:                Compute $w_{j,next}$$1\leq j\leq N_{rn}$51:                **if** $w_{cr,next}<w_{0_{cr,next}}$ **then**52:                    imp←153:                **else**54:                    Restore previous state55:                    trial←trial+156:                    **if** imp=1 **then** imp←057:                    **else**58:                        **if** trial>2 **then**59:                            imp←060:                        **else**61:                            ek←162:                        **end if**63:                    **end if**64:                **end if**65:            **else**66:                imp←067:            **end if**68:        **end while**69:    **end if**70:    imp←171:____________________________________________________________________________________________________
    **Distance optimisation**72:____________________________________________________________________________________________________
73:    trials←074:    **while** $\left(improve=1\right)\;\&\;(w_{cr,next}>1+\zeta )$ **do**75:        Store previous state ( $PL_{0}\leftarrow PL$, $d_{0}^{2D}\leftarrow d^{2D}$, $d_{0}^{3D}\leftarrow d^{3D}$)76:        $\Delta d\leftarrow d_{0_{cr,next}}^{2D}*\rho$77: 78:        **if** $d_{0_{cr,next}}^{2D}>0$ **then**79:            $\Omega \leftarrow ∠(tx_{cr}.tx_{next})$80:            $\phi_{next}\leftarrow F\left(\phi_{0},d_{0_{cr,next}}^{2D}-\Delta d,\Omega \right)$ {new latitude}81:            $\lambda_{next}\leftarrow G\left(\lambda_{0},d_{0_{cr,next}}^{2D}-\Delta d,\Omega \right)$ {new longitude}82:            Compute $w_{cr,next}$83:            **if** $w_{cr,next}<=w_{0_{cr,next}}$ **then**84:                imp←185:            **else**86:                **if** $d_{0_{cr,next}}^{2D}>d_{cr,next}^{2D}>$ **then**87:                    **if** trials<nt **then** {Avoid values where PL is constant, nt, number of trials}88:                        trial←trial+189: 90:                        **continue**91:                    **end if**92:                **else**93:                    imp←094:                    Restore previous state95:                **end if**96:            **end if**97:        **else**98:            imp←099:        **end if**100:    **end while**101:    P← shortest path nodes between GW and target WSN102:    cr←CN[k]103:    k←k+1104:    **if** cr=CN[end] **then**
105:        **break**106:    **end if**107:
**end while**
108:$\phi_{opt,n_{i}}\leftarrow \phi_{n_{i}}$, $\lambda_{opt,n_{i}}\leftarrow \lambda_{n_{i}}$, $\epsilon_{opt,n_{i}}\leftarrow h_{n_{i}}$109:
**return**
110:

$\phi_{opt,n_{i}},\lambda_{opt,n_{i}},\epsilon_{opt,n_{i}}$





## 7. Positioning Algorithm Evaluation and Results

Algorithm 2 was implemented using MATLAB R2019b IDE and executed on a six-core processor, 12 logical processor station. The implemented code was profiled to evaluate the potential improvement of its performance. For example, only the call of built-in MATLAB “distance” function for geodesic distance increased with an algorithm execution time over 53 s. After implementing our own geodesic distance computing function based on the Haversine Equation [80], the elapsed time decreased from more than 140 s to less than 16 s.

The values of $a_{j}$ and $b_{j}$ in Equation (28) are equal to 1.

**Figure 11:**
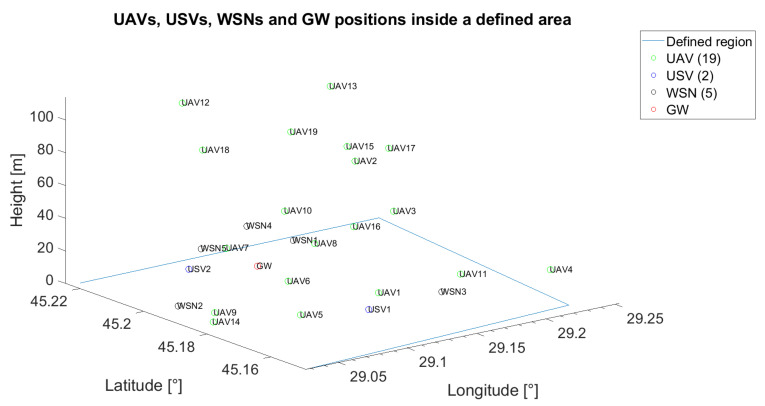
Initial positions before applying the algorithm.

The initial placement for all the following scenarios is depicted in Figure 11, whereas the target wireless sensor network is WSN4.

We introduce the performance indicator σ, which represents the ratio between the sum of all subunitary weights of the edges of the path and the total number of edges of the path of the communication graph ($\sigma =\frac{s}{n_{vn}+1}$, where *s* is computed based on Equation (30). When a successful link is established between the gateway and served WSN, $s\to n_{vn}+1$ and, thus, σ→1. The significance of very low values of σ and $\sum_{i=0}^{n_{vn}}c_{i}=n_{vn}+1$ would reside in the fact that more close UVs than necessary are acting as relays, rising interference issues and inefficient placement or UV choice, which is not the case for the evaluated scenarios.

(30) $$s=\sum_{i=0}^{n_{vn}}\frac{c_{i}PL_{CN[i],CN[i+1]}}{LB}$$

where CN[0] represents the gateway, $CN[n_{vn}+1]$ represents the served WSN and

(31) $$c_{i}=\left\{\begin{array}{cc} 1 & PL_{CN[i],CN[i+1]}\leq LB\\ 0 & PL_{CN[i],CN[i+1]}>LB \end{array}\right.$$

Further, we denoted the distance between gateway and WSN ($d_{WSN-GW}$) by *d*. The number of necessary and sufficient UVs ($n_{UAV}$, $n_{USV}$) and virtual nodes, $n_{vn}$, for each scenario was determined based on the simulations, that is, we determined the minimum values for which the communication link for gateway–served WSN can be ensured.

### 7.1. Algorithm Evaluation for LB=92 dB

In this subsection, we present the algorithm evaluation for the technical solution in [58] (Wi-Fi, LB=92 dB) for three scenarios, with three different values of the distance between the gateway and the served WSN (d=2.393 km, d=7.2209 km, d=9.3362 km). We assumed that the investigated propagation scenario is Rural Macro-Cell, for which the maximum radius is 10 km. Therefore, the maximum distance between the served WSN and GW for which we performed the simulations did not exceed this value.

#### 7.1.1. Algorithm Results for d=2.393 km

We consider the first scenario for the technical solution having LB=92 dB. The distance between the gateway and the served WSN is d=2.393. The number of UAVs equals 19, whereas the number of USVs is 2. The number of virtual nodes implied in the scenario is seven. That is, in this scenario, only seven UVs need to modify their position to ensure the communication link between the gateway and the served WSN. Table 10 presents the evaluation results in terms of execution time and performance indicator σ.

**Figure 12:**
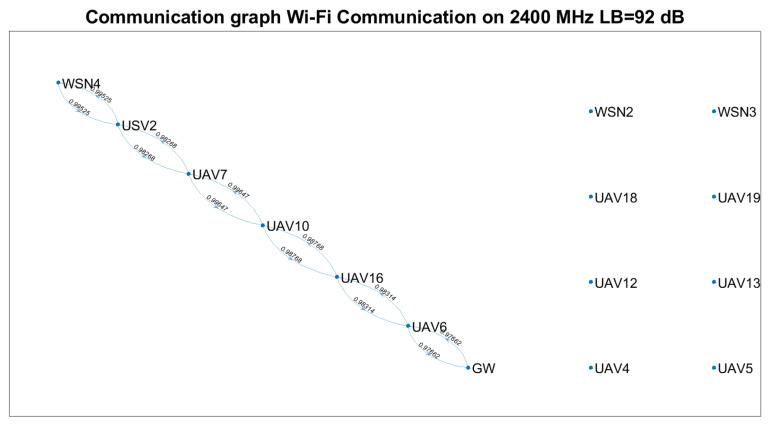
Algorithm evaluation: Communication chain and corresponding weights. δd=20 m, δh=7 m, ρ=0.2, LB=92 dB, d=2.393, $n_{vn}=7$.

In Figure 12, we present the entire communication chain, GW–UVs–WSN, with the corresponding UVs that ensure the communication between the two points for the scenario illustrated in Table 10. All edges have subunitary weights, and the weights are very close to 1, which indicates that there are not more close UVs acting as relays than required and that they are correspondingly placed. In addition, the representation emphasises that only 5 out of 21 UVs and 5 out of 7 virtual nodes are necessary to ensure the communication link.

**Figure 13:**
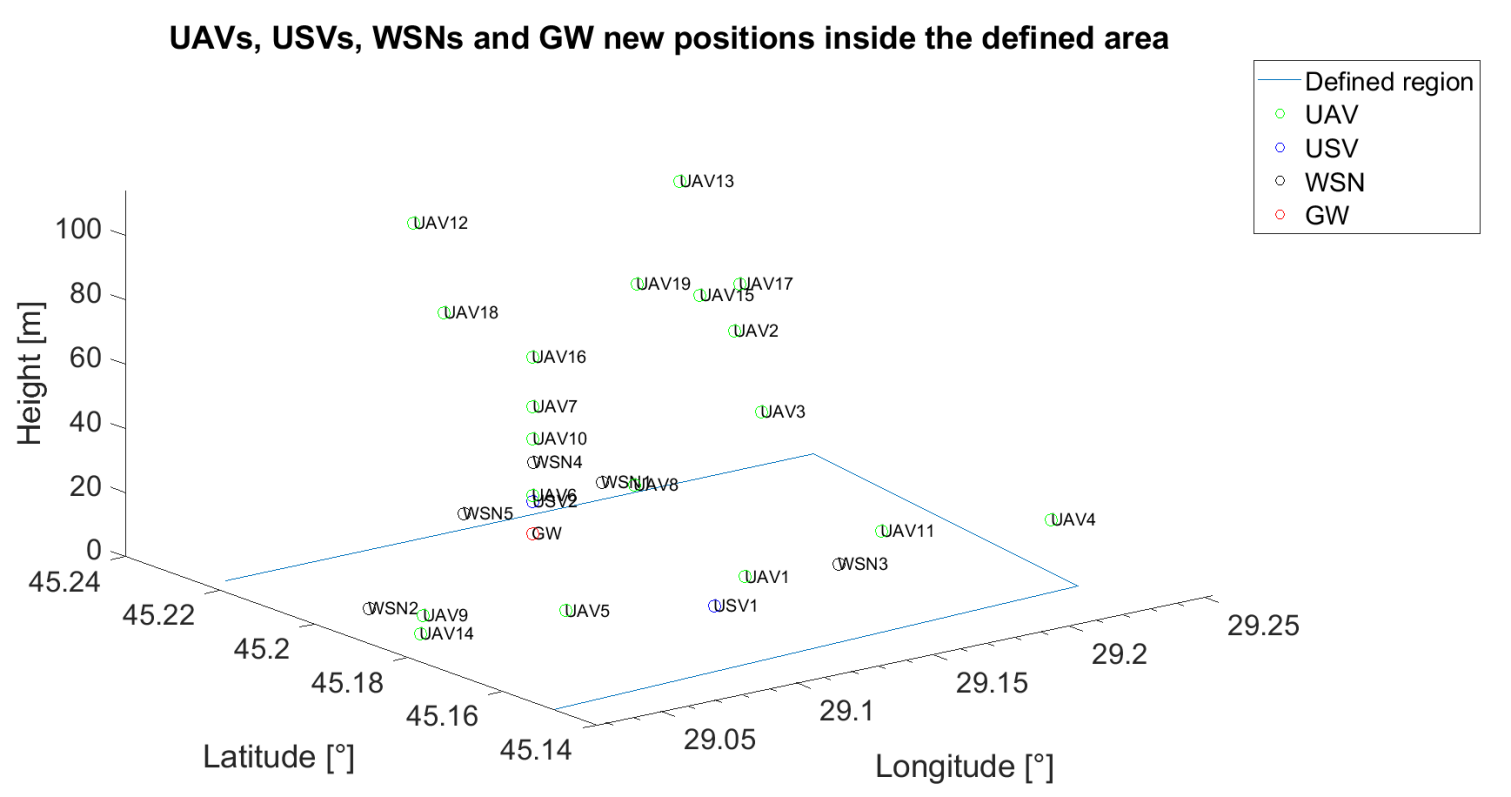
Final positions after applying the algorithm. Scenario depicted in Table 10, ρ=0.2.

Figure 13 presents the final positions of the UVs for the corresponding communication graph in Figure 12 with the results presented in Table 10. The parameters were: δd=20 m, δh=7 m, ρ=0.2 LB=92 dB, d=2.393 km and $n_{vn}=7$.

#### 7.1.2. Algorithm Results for d=7.2209 km

A new scenario targets the increase in the distance between the gateway and the served WSN to d=7.2209. For the same number of UAVs and USVs (19 and 2, respectively) and the same technical solution of LB=92 dB as in previous scenario, the number of virtual nodes that would cover this new larger distance must increase to 19. Table 11 presents the evaluation of the algorithm.

To highlight the improvements made after profiling the implemented code, we included the time execution before profiling ($t_{1}$) and after the improvements ($t_{2}$) in Table 11. Τhe execution time for Algorithm 1 for the scenario presented in Table 11 is 0.0418 s, while the positioning algorithm requires between 8.982 s and 24.073 s to be executed.

We notice that the best results for σ are obtained for ρ=0.1 and the execution time $t_{2}=14.347$. Nevertheless, a good tradeoff is ρ=0.2, where the execution time decreases to 8.982 s.

The cases where σ is marked with * are for unsuccessful link $\sum_{i=0}^{n_{vn}}c_{i}\neq n_{vn}+1$.

Comparing the results corresponding to the scenarios proposed in Table 10 and Table 11, one can notice that the best value of distance step for these scenarios remains ρ=0.2.

#### 7.1.3. Algorithm Results for d=9.3362 km

The third scenario envisions the behaviour of a similar network where the distance between the gateway and the served WSN increases to 9.3362 km. The number of available UAVs and USVs must be increased. Furthermore, the number of virtual nodes increases to 28 in order to cover the entire distance. Table 12 reflects the evaluation results. By comparing the results obtained in Table 11 and Table 12, one remarks that the distance step parameter ρ=0.12 should be avoided for both scenarios, whereas for the scenario in Table 12, ρ should not exceed 0.1.

### 7.2. Algorithm Evaluation for LB = 106 dB and LB = 115 dB

Further, we evaluate the algorithm when one of the technical solutions in [57] (long range, LB=106 dB) and [59] (LoRa/LoRaWAN, LB=115 dB) is used. The same distances, d=2.393 km, d=7.2209 km and d=7.2209 km, were investigated.

#### 7.2.1. Algorithm Evaluation for LB=106 dB and LB=115 dB when d=2.393 km or d=7.2209 km

For evaluating the technical solutions in [57,59], we considered the simulation parameters in Table 13. The values are identical with the ones used for the technical solution in [58] (for comparison, see Section 7.1.1 and Section 7.1.2, Table 10 and Table 11). The technical solution based on long range communication [57] succeeds in assuring the communication link without any improvements from the point of view of UV placement (without it being necessary to apply the algorithm and to move the UVs from their initial positions). The same is valid for the solution in [59], both being candidates to ensure the communication link between GW and any of the UVs while transmitting their GPS coordinates.

#### 7.2.2. Algorithm Evaluation for *LB* = 106 dB, d=9.3362 km

**Figure 14:**
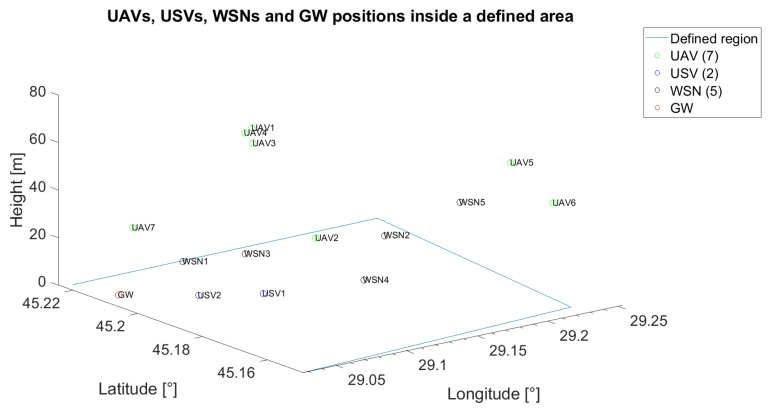
Initial positions. Scenario depicted in Table 14, ρ=0.2.

In this subsection, another scenario was investigated for the technical solution in [57], when the distance between gateway and target WSN is 9.3362 km. It is different from the previous ones proposed for lower *LB*, because the coverage range for the solution in [57] is higher and, thus, a lower number of UVs would cover the distance between the GW and the served WSN. Consequently, we propose an initial placement of seven UAVs, two USVs and five WSNs, as depicted in Figure 14.

**Figure 15:**
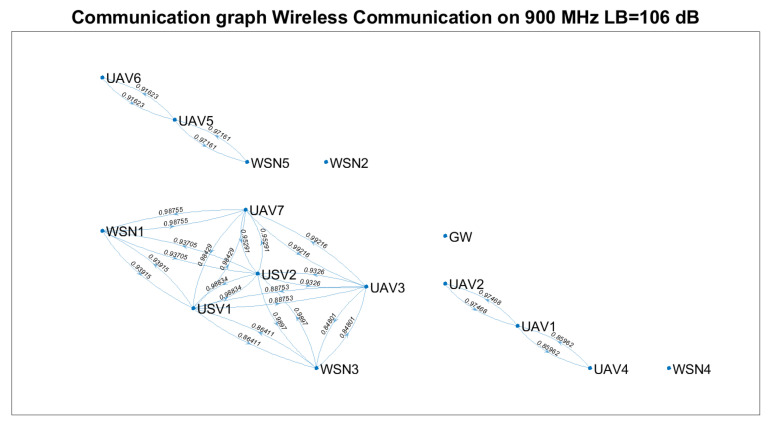
Corresponding initial communication graph. Scenario depicted in Table 14, ρ=0.2.

The corresponding initial communication graph is given in Figure 15.

For this scenario, the simulation parameters and the evaluation results are given in Table 14.

#### 7.2.3. Algorithm Evaluation for *LB* = 115 dB, d=9.3362 km

We repeated the simulations for the scenario in Section 7.2.2, this time for the technical solution in [59] having LB=115 dB. The simulation parameters are the same ($n_{UAV}=7$, $n_{USV}=2$, $n_{vn}=7$). We observed that no placement solution was identified.

Further, we increased the number of virtual nodes ($n_{vn}$ = 8) and the same behaviour was noticed. Finally, for $n_{vn}$ = 9 a solution, as seen in Table 15, was determined.

From Table 15, we can state that there are very low values of σ. Because $\sum_{i=0}^{n_{vn}}c_{i}=n_{vn}+1$ (communication link was ensured), more close UVs act as relays than needed.

Given all the above, the proposed algorithm is less suitable for networks equipped with high *LB* communication equipment. Even though apparently this represents a drawback, we recall that the algorithm was intended for determining the optimum position of the UVs nodes such that one allows a fast data transfer between a served WSN and a GW when a shorter-range, high-throughput communication technology is employed, a scenario in which the algorithm has very good performance.

### 7.3. Discussion

In addition to the remarks mentioned in the previous subsections, other essential aspects deserve our attention. They refer to (1) the real-time applications vs. non-real-time ones and to (2) the mobility states between initial and final positions. Concerning the first aspect, the monitoring tasks assigned by the Cloud Center are not critical ones, as usually, the sampling time in environment monitoring takes minutes or even hours to reduce the power consumption of the devices [81]. An execution time of 8.982 s is sufficient for these purposes, even though the implementation can be further enhanced to reach less than 4 s execution time. Concerning the mobility states of the UV between CC trigger–UV initial position sending–CC algorithm execution–UV final positions sending, this can be mitigated by forcing the UVs to keep their positions between the trigger and the CC position message sending time window. Without such mitigation measure, for a theoretical horizontal speed of 16 m/s, the new position of a drone may change with 320 m for a time window of 20 s. For a theoretical vertical speed of 6.28 m/s, the summed vertical offset (for consecutive up-down movements) can vary over 120 m.

## 8. LoRa/LoRaWAN/Wi-Fi Use Cases

### 8.1. USV: Ship Obstacle Avoidance

On the channels of the Danube Delta, silting frequently appears as a consequence of the water level decrease. The installation of classical guiding buoys represents a solution to avoiding the affected regions. However, they do not cover the entire affected area or are not installed in time when the water level is modifed. A solution consists of using autonomous aquatic vehicles with small dimensions, equipped with GPS modules and ultrasonic sensors for constant monitoring of the navigation channels and mapping the underwater land-forms under continuous change. The same vehicles are also able to identify obstacles, such as fallen trees etc. For improved autonomy, these vehicles can be connected by employing the LoRa communications technology.

### 8.2. USV: Monitoring Water and Environment Quality

The architecture can be expanded to monitor the quality of water and the parameters of the environment by the aggregation of the data from the sensors on the ground, as well as from sensors that can be placed on the boats of local people. In this use case, the Danube Delta administration or the associations of fishermen can be beneficiaries. The boats of local people can thus become nodes for collecting data regarding the environment.

### 8.3. USV+UAV: Ensuring an Ad Hoc Data Link for Devices in Faraway Regions of the Delta

Boats and drones can act as temporary access points to ensure a data link between some access regions. For example, local people who possess agricultural lands monitored using agricultural sensors can remotely verify the status of parcels using an ad hoc network created when the boats pass through the area covered by the wireless sensors network for smart agriculture.

## 9. Conclusions

Through this paper, we emphasised the impact of UAV and USV networks in the Internet of Things ecosystem, and we explored the concept of Internet of Unmanned Vehicles as an extrapolation of the concept of Internet of Drones (or Internet of Unmanned Aerial Vehicles), which was narrower. In addition, we proposed an IoT architecture based on a hybrid network of WSNs, UAVs and USVs (UVs) that have two main roles: (1) to ensure communication between a served WSN and the gateway and (2) to fulfil tasks assigned by a Cloud Center (CC).

For the UVs to achieve the first role, we presented the background for the propagation models employed to model the communication link between aerial, aquatic and terrestrial nodes. Based on the theoretical study, we implemented two algorithms that can run on a Cloud server hosted by the Cloud Center. The first one uses, for the first time according to our knowledge, the orthodrome (great circle path) concept to reduce the optimum location searching space and, thus, the complexity of the position optimisation algorithm, which is the second one proposed in the paper. The first algorithm successfully determines the closest UV nodes to specific virtual nodes on the orthodrome. The proposed algorithm gives very good results and it is not influenced by the distance between the gateway and the served WSN. Instead, its execution time is influenced by the number of real nodes (UVs). Nevertheless, for a hypothetical number of 100 UVs, the output of the algorithm is returned in around 0.116 s. After applying the great circle method and its corresponding algorithm, the placement optimisation algorithm determines the number and the optimum positions of UVs such that the communication between the served WSN and gateway is ensured. We proposed concepts such as communication graphs and metrics as the weight of the graph and a performance indicator, σ. The latter reveals whether there are more UVs acting as access points than needed or not when the communication is ensured. The algorithm was implemented in MATLAB 2019b. The set of pseudorandom initial coordinates of UVs and the static coordinates of the gateway and of the WSNs correspond to a region in the Danube Delta, but the algorithm is not restricted to this area alone. The proposed optimisation algorithm uses an adaptive step for the distance, reducing its execution time. For example, in the case of a Wi-Fi-based technical solution, the execution time decreases from 9.154 s to 3.285 s when an adaptive distance coefficient ρ=0.2 is used and the distance between the gateway and the served WSN is 2.393 km. When the distance increases to 7.2209 km, the usage of the same adaptive distance coefficient determines the decreasing of the execution time from 24.073 s to 8.982 s. In what concerns the performances of the placement optimisation algorithm when LoRa/LoRaWAN technology is used, on the one hand, we revealed that the algorithm is not necessary for the distances of 2.393 and 7.2209 km tested for Wi-Fi communication. On the other hand, when a technical solution having LB=106 dB is used, the performance indicator of the algorithm decreases, while the execution time increases to 44 s. Even lower performances (high execution time, more UVs acting as relay than needed) are observed when using the technical solution with LB=115. Nevertheless, the goal of the algorithm resides in providing the optimum positions of the UVs such that fast data transfer occurs when low *LB*, high-throughput communication technology such as Wi-Fi is employed. Additionally, if needed, based on the positions computed and on the UVs’ equipment (sensors, power supply etc.), the Cloud Center can adaptively assign specific monitoring tasks to UVs.

Afterwards, we highlighted the main use cases arising from the proposed IoT architecture and algorithm when LoRa/LoRaWAN communication technology alone is employed or when LoRa/LoRaWAN, Wi-Fi and cellular communications are used in the Danube Delta developing region. Nevertheless, the use cases can be extended to other near-to-water regions.

### 9.1. Comparison with Other Works

We outline a comparison between other works in the domain and our proposed approach. As compared to [11] where two separate gateways are used (one for UAVs fleet and one for USVs fleet), our architecture uses a simpler approach with only one gateway for both UAVs and USVs. Moreover, their analysis focuses on the routing protocols and network capacity, not on channel propagation. In contrast to [44] where a path planning algorithm based on environmental parameters (wind speed and direction) for UAVs is presented, we propose an architecture and a positioning algorithm based on the communication parameters related to path loss that allows the positioning of UVs to deploy an entire network infrastructure based on UAVs and USVs. With respect to [45,46], our algorithm uses all types of UAV channels (A2A, A2G equivalent to G2A) and USV channels (OW, S2S, A2S). In addition, our algorithm does not employ only one UAV as in [45]. In comparison with [46] where the air-to-air channel models are investigated, we used UAV propagation models adapted to a wider range of applications. For example, concerning UAV-related channels, besides the air-to-air channel, we analysed an air-to-ground channel (having the same properties as the ground-to-air channel). We drew the conclusion that the 3GPP model for Rural Macro-cell is very suitable for isolated areas, with poor coverage as those covered by the current paper (e.g., the Danube Delta). With respect to [47,48,49,50,51], our proposed algorithm is not intended for USV path planning but for finding the optimum final positions of the UV nodes that ensure the formation of a USV–UAV network in conditions of communication reliability.

To conclude this subsection, according to our knowledge, our proposed approach is unique. It can be the starting point for many collaborations between specialists in the different layers of Internet of Things architecture to build a strong Internet of Unmanned Vehicles ecosystem.

### 9.2. Future Perspectives and Open Challenges

We observed that the propagation models in the literature investigated and employed in the simulations of the current paper have limitations that render them not applicable in all real-life scenarios. Consequently, we intend to explore new channel models to reach beyond the limits of the propagation models employed until now.

The proposed algorithm has advantages and limitations. An advantage of the algorithm consists of the scalability in terms of the propagation models, UVs initial positions and other parameters. Consequently, further, we would like to implement more complex propagation models. In the future, we will enhance the algorithm to consider the impact of the interference experienced by UAVs at low altitude [82]. Based on the proposed algorithm and the implementation of collision avoidance techniques [83], an autopilot system can be further enhanced.

A drawback of the algorithm, which will be mitigated in future, consists of the high execution time for technical solutions involving a higher link budget.

Finally, we will perform thorough measurement campaigns and define propagation loss models based on on-field experimental measurements.

## Figures and Tables

**Table 1:** Parameters of transceivers involved in the simulations.

Parameter	Description	[57]	[58]	[59]
f (MHz)	Frequency	900	2400	868
$P_{tx}$ (dBm)	Maximum Transmitter power	20	14	14
$S_{rx}$ (dBm)	Sensitivity	−106	−98	−121
LB (dB)	Final Link Budget	106	92	115
DR	Data Rate	115 kbps	72 Mbps	37.5 kbps
**Technology**		Long Range	Wi-Fi	LoRa

**Table 2:** Comparison between LoRa on 868 MHz and LoRa on 433 MHz.

Parameter	868 MHz	433 MHz
Maximum allowed TX power	14 dBm	10 dBm
Antenna’s dimension	L	2× L
Congested	no	yes
Packet reception stability [60]	yes	no
Penetration capability [60]	no	yes
Overall performances [61]	Better at the edge of the coverage area	Better close to the GW

**Table 3:** UAV-to-ground models using 3GPP.

Model	Usage
RMa	Large areas with radii up to 10 km and low building densities; BS antenna > average buildings height [70]
UMa	BS antenna above buildings heights, NLoS or obstructed-LoS, BS–BS distance < 3 km [71]
UMi	Higher LoS probability than UMa [70], BS–BS distance < 1 km [71]

**Table 4:** Parameters involved in Equations (7)–(10): description and measure units.

Parameter	Description	Measure Unit
f	Frequency	Hz
d	Geodesic distance between transmitter and receiver	m
$h_{t}$	Height of transmitter’s antenna	m
$h_{r}$	Height of receiver’s antenna	m
$h_{d}$	Height of duct	m
c	Light speed in vacuum	m/s

**Table 5:** Values of the parameters involved in simulations based on Equations (7)–(10): description and measure units.

Parameter	Value	Step
f	900 MHz	-
d	1–10,000 m	50 m
$h_{t}$ (USV)	0.5–5 m	0.5 m
$h_{r}$ (UAV)	5–120 m	20 m
c	$3\times 10^{8}$ m/s	-

**Table 6:** Examples of recommendations for the scenario when $h_{t}=5$ m, *LB* = 106 dB.

$h_{t}\left(m\right)$	$h_{r}\left(m\right)$	Recommendations
5	5	d < 1219 m
	25	d≠ 235 m; d < 2777 m
	45	d≠ 217 m; d≠ 423 m; d < 3714 m
	65	d≠ 154 m; d≠ 310 m; d≠ 621 m; d < 4472 m
	85	d≠ 115 m; d≠ 205 m; d≠ 405 m; d∉ [800,825] m; d < 4817 m
	105	d≠ 167 m; d≠ 250 m; d≠ 329–341 m; d≠ 497 m; d∉ [967,1053] m; d < 5520 m

**Table 7:** Proposed environment, channels, scenarios and path loss models for aerial, terrestrial and water communications.

Environment	Channel	Scenario	PL Model
Rural	A2G	UAV-GW UAV-WSN	3GPP
	A2A	UAV-UAV	3GPP (h<60 m) FSPL (h≥60 m)
	G2G	WSN-GW WSN-WSN	3GPP
	S2S	USV-GW USV-WSN	2-ray
	OW	USV-USV	
	A2S	UAV-USV	

**Table 8:** Communication graph-based evaluation of technical solutions in [57,58,59].

KPI	[57]	[58]	[59]
Number of connected nodes	11	2	12
Number of nodes connected to GW	1	0	12
Number of edges	20	2	36
$w_{min}$	0.75781	0.96573	0.69577
$w_{max}$	0.99934	-	0.98583

**Table 9:** Path division algorithm results: execution time with respect to distance between GW and WSN and number of UVs.

$n_{\mathrm{UAV}}+n_{\mathrm{USV}}$	*d* (km)	*t* (s)
3	1.0	0.0348
	1.5	0.0325
	2.0	0.0343
10	1.5	0.0323
	2.0	0.0404
	5.0	0.0295
	8.0	0.0325
20	2.0	0.0377
	5.0	0.0295
	8.0	0.0322
52	2.0	0.0493
	5.0	0.0427
100	2.0	0.1152
	5.0	0.1165

**Table 10:** Algorithm results: execution time (after time execution improvement) and performance indicator σ (nt=3, LB=92 dB, d=2.393 km).

$n_{\mathrm{UAV}}$	$n_{\mathrm{USV}}$	$n_{\mathrm{vn}}$	*d* (km)	ρ	δd (m)	$t_{2}$ (s)	σ
19	2	7	2.393	0.05	20	9.154	**0.9929**
				0.1	20	8.851	0.9711
				0.12	20	4.569	0.9895
				0.13	20	4.716	0.9902
				0.14	20	3.857	0.9888
				0.15	20	7.625	0.9665
				0.2	20	**3.285**	0.9870

**Table 11:** Algorithm results: execution time (before and after the improvement) and performance indicator σ (nt=3, LB=92 dB, d=7.2209 km).

$n_{\mathrm{UAV}}$	$n_{\mathrm{USV}}$	$n_{\mathrm{vn}}$	*d* (km)	ρ	δd (m)	$t_{1}$ (s)	$t_{2}$ (s)	σ
19	2	19	7.2209	0.05	20	149.29	24.073	0.9859
				0.1	20	113.649	14.347	**0.9871**
				0.12 *	20	126.498	14.424	0.9347 *
				0.13	20	115.96	12.913	0.9836
				0.14	20	110.018	12.008	0.9813
				0.15	20	120.43	11.118	0.9815
				0.2	20	116.178	**8.982**	0.9830

**Table 12:** Algorithm results: execution time (after time execution improvement) and performance indicator σ, nt=3, LB=92 dB, d=9.3362 km.

$n_{\mathrm{UAV}}$	$n_{\mathrm{USV}}$	$n_{\mathrm{vn}}$	*d* (km)	ρ	δd (m)	$t_{2}$ (s)	σ
30	2	28	9.3362	0.05	20	36.825	0.9821
				0.1	20	22.875	**0.9887**
				0.12 *	20	20.203	0.9550 *
				0.13 *	20	19.875	0.9533 *
				0.14 *	20	18.383	0.9543 *
				0.15 *	20	18.465	0.9528 *
				0.2 *	20	**14.774**	0.9525 *

**Table 13:** Parameters used in the simulations for *LB* = 106 dB and *LB* = 115 dB.

$n_{\mathrm{UAV}}$	$n_{\mathrm{USV}}$	$n_{\mathrm{vn}}$	*d* (km)
19	2	7	2.393
19	2	19	7.2209

**Table 14:** Algorithm results: execution time (after time execution improvement) and performance indicator σ (nt=3, LB=106 dB, d=9.3362 km).

$n_{\mathrm{UAV}}$	$n_{\mathrm{USV}}$	$n_{\mathrm{vn}}$	*d* (km)	ρ	δd (m)	$t_{2}$ (s)	σ
7	2	7	9.3362	0.05	20	45.132	0.9634
				0.1	20	46.067	0.9589
				0.12	20	44.819	0.9571
				0.13	20	44.248	0.9561
				0.14	20	44.159	0.9552
				0.15 *	20	44.119	0.9542 *
				0.2 *	20	44.089	0.9525 *

**Table 15:** Algorithm results: execution time (after time execution improvement) and performance indicator σ (nt=3, LB=115 dB, d=9.3362 km).

$n_{\mathrm{UAV}}$	$n_{\mathrm{USV}}$	$n_{\mathrm{vn}}$	*d* (km)	ρ	δd (m)	$t_{2}$ (s)	σ
7	2	9	9.3362	0.05	20	32.4113	0.8807
				0.1	20	32.1097	0.8839
				0.12	20	31.9687	0.8783
				0.13	20	32.0568	0.8792
				0.14	20	32.1442	0.8801
				0.15 *	20	31.9802	0.8809
				0.2 *	20	31.8643	0.8844

## Data Availability

The data presented in this study are available on request from the corresponding author.

## Abbreviations

The following abbreviations are used in this manuscript:

3D	tri-dimensional
3GPP	3rd Generation Partnership Project
A2A	Air to air
A2G	Air to ground
A2S	Air to ship
CC	Cloud Center
DR	Data Rate
FSANET	Flying and Surface Ad Hoc Network
FSPL	Freespace Path Loss
G2G	Ground to ground
GCS	Ground Control Station
GW	Gateway
GW-UVs-WSN	Gateway–Unmanned Vehicles–Wireless Sensor Network
GRS	Ground Recharging Station
ITU	International Telecommunication Union
LB	Link Budget
LoS	Line-of-Sight
NLoS	Non-Line-of-Sight
NLP	Non-linear Programming
OW	Over-water
PL	Path Loss
RX	Receiver
S2S	Ship to Shore
TX	Transmitter
UAV	Unmanned Aerial Vehicle
UHF	Ultra High-Frequency
USV	Unmanned Surface Vehicle
UV	Unmanned Vehicle
VHF	Very High-Frequency
WSN	Wireless Sensors Network
